# Continuous monitoring of soybean (*Glycine max*) electrical signaling during stink bug (*Euschistus heros*) infestation and plant protection measures

**DOI:** 10.1038/s41598-025-25530-2

**Published:** 2025-11-24

**Authors:** Jurrian Friedrich, Andrzej Kurenda, Nicole Furrer, Thorben Müller, Keith Ward, Anke Buchholz

**Affiliations:** 1 Syngenta Crop Protection AG, Schaffhauserstrasse 101, 4332 Stein, Switzerland; 2 Vivent SA, Rue Mauverney 28, 1196 Gland, Switzerland; 3 Syngenta Ltd, Jealott’s Hill International Research Centre, Bracknell, Berkshire RG42 6EY UK

**Keywords:** Plant electrophysiology, Plant–insect interaction, Plant stress, Plinazolin-technology, Verdavis, Ecology, Ecology, Plant sciences

## Abstract

Stink bug feeding on soybean pods requires vigilant crop protection due to low economic thresholds. This study used extracellular plant electrophysiology (EPE) to continuously monitor plant electrical signals (ES) during infestation. Greenhouse experiments evaluated crop infestation scenarios with the insecticide Verdavis (isocycloseram and lambda-cyhalothrin). Plant ES were recorded for different infestation periods, and related crop damage was compared with EPE output. Based on these experiments, an EPE model was developed to quantify spike occurrences. Distinct spike counts emerged within the first day of infestation: infested plants with visual damage after 7 days generated significantly more ES spikes compared to non-infested controls (*p* < 0.001). Conversely, plants that showed only marginal damage after 7 days did not exhibit significant differences in ES spike generation (*p* = 0.058). Treated plants generated similar spike patterns as non-infested plants, indicating effective plant protection. Manual insect removal led to rapid reduction of spike count. Additionally, more spikes were recorded during the day (*p* < 0.001), but spike output was similar between pod- and stem-inserted electrodes during day (*p* = 0.837) and night (*p* = 0.328). This study demonstrates that EPE can be used as a minimally invasive method for real-time monitoring of plant protection, providing insights into plant–insect interactions and insect control measures.

## Introduction

Soybean (*Glycine max*) accounts for roughly half of the world’s oilseed and plant-based protein production^1^. In the 2023/2024 season, the top three producers, Brazil, the USA, and Argentina, contributed 39%, 29% and 12% respectively to the global 395 million metric tons^2^. As global population and meat consumption rise, soybean demand is expected to increase in the coming years^3^. However, production faces challenges, with global annual yield losses due to pests and pathogens in soybean estimated at 21.4%^4^.

The introduction of Bt-soybean drastically reduced infestation levels of main lepidopteran pests like *Chrysodeixis includens* and *Anticarsia gemmatalis* (Lepidoptera: Noctuidae)^5^. However, stink bugs (Hemiptera: Pentatomidae) continue to represent a serious economic threat to various agricultural crops^6^. On soybeans, they are mainly pod feeders and infestation can result in yield loss, reduced soybean quality and delayed ripening, posing a significant threat for growers^7,8^. Due to this pod-feeding behavior, the economic damage threshold is as low as 1 to 2 individuals per row meter^9^. If left unchecked, infestation can increase to 40 to 80 individuals per row meter in late season^9^. Especially due to late-season population migrations from soybean fields, stink bugs also inflict considerable harm on neighboring cotton (*Gossypium* spp.) and corn (*Zea mays*) fields^9^.

Over recent decades, the highly competitive neotropical brown stink bug (*Euschistus heros*) has emerged as the most prevalent stink bug species in soybean fields in central and south America^6,10^. Between 2015 and 2017, this species accounted for 64% to 82% of all stink bugs collected in soybean fields^9^. Together with several reported cases of stink bug resistance to pesticides, this can create a vicious circle of pesticide mismanagement that results in a concerning situation for future soybean production^11^.

Stink bug feeding necessitates fast plant protection. When feeding, the insect injects extraoral saliva enzymes into the plant, which causes damage that perpetuates post infestation^12^. Moreover, the discontinuous feeding style and distinct feeding strategies make stink bugs difficult to study. When feeding on local mesophyll tissue, feeding consists of a combination of mechanical (laceration) and chemical (maceration) steps, i.e., cell rupture strategies^13^. Whereas feeding on vascular tissue is characterized by the formation of a lubricating and anchoring salivary sheath (salivary sheath strategy)^13^.

Besides being dynamically complex, feeding is almost imperceptible to the naked eye. Conventional assessments of agronomical studies are mainly limited to counting the presence of stink bugs and doing endpoint crop damage assessments. Continuous assessments of stink bug feeding patterns are predominantly confined to electropenetrography (EPG) studies. This technique provides high-resolution insights into the complexity of stink bug feeding mechanisms^13^. However, the mandatory use of a Faraday cage and the limitation of recording one insect at a time limit the ecological and agronomical validity of these studies in simulating field-realistic infestation scenarios.

An alternative methodological approach to creating a closed electric circuit between insects and plants is to monitor endogenous bioelectrical signals generated by plants in response to herbivory. The focus thereby shifts from insect feeding to induced plant stress. Furthermore, measuring plant electrical signals (ES) during stink bug infestation would help to understand how information about the infestation is transmitted throughout the plant and to what extent physical and chemical stimuli provoke signal transmission.

Plants generate ES in response to environmental stimuli like cold, heat or wounding^14^. Although plants do not have neurons like animals, they have developed unique ways to produce ES and efficiently transport these over long distances. Cell-to-cell propagation is slower between plant cells than along neurons^15^. However, plants make effective use of minute hydraulic pressure waves in the vascular system and convert these into electrical signals in distal tissues through mechanosensitive calcium channels^16^. Plant ES have been categorized into different groups based on their waveform properties and mode of propagation^17^.

On a cellular level, transmembrane depolarization is currently thought to be one of the first signaling events in the plant’s response cascade. This process involves cytosolic calcium influx, which then triggers reactive oxygen and nitrogen species production^18^. Electrosensitive calcium channels provoke a chain reaction of depolarized cells, ultimately inducing distal stress responses^19,20^. The type of ES determines the subsequent alterations in gene expression, leading to various downstream responses, including phytohormone production and changes to photosynthesis and respiration^21,22^.

Plants face a diverse array of organisms that feed upon them and have consequently evolved sophisticated mechanisms to discriminate between distinct types of attack. Plants can differentiate between singular and sustained mechanical damage, as well as distinguish the presence of insect saliva^23^. As a result, these stimuli trigger distinct electrical signal cascades within the plant. This fact can be exploited in studies with stink bug infestations, as their stylet punctures plant tissues and releases extraoral digestive enzymes into the plant.

Several techniques can be used to measure ES in plants. They differ in their approach, sensitivity, and applicability to different experimental conditions, each offering unique advantages and limitations^24^. Of these, extracellular plant electrophysiology (EPE) enables experimental setups that are closest to real-world conditions. It is a minimally invasive technique that involves the insertion of metal microelectrodes into plant tissues for bioelectrical potential measurements.

EPE enables continuous recording of plant ES over multiple weeks^25^. Furthermore, data collection of plant responses does not require a Farraday cage to block background signals. Nevertheless, the measured target tissue is unspecific, consisting of the total electrical field surrounding the electrode surface. Electrode insertion also causes initial wounding responses due to mechanical damage. However, the comparison of surface electrodes (applicable for short-term recordings) and piercing electrodes after initial wound recovery, revealed essentially identical recordings^26^.

Deciphering ES that are specifically induced by herbivory, a plant’s response can be measured in complex experimental settings^27^. The advantages of plant electrophysiology become particularly evident when monitoring herbivore presence or their feeding, when this is otherwise difficult to detect. This was exemplified in a study where a supervised machine learning model was created for plant stress in response to root-knot nematode (*Meloidogyne incognita*) infection^28^.

Although stink bugs are easy to recognize by eye it is hard to detect their feeding activity in real time, and their infestation behavior changes over time. Identifying plant ES that correlate with stink bug infestation and plant damage would facilitate monitoring and help understanding stink bug behavioral dynamics.

In this study we continuously monitored soybean plants for several days using EPE while they were infested with *E. heros.* Our aim was to develop a mathematical plant ES stress model that correlates with infestation intensity. The research comprises three main experiments, all conducted on potted plants under greenhouse conditions. The first two experiments focused on plant ES response in early- and late-season infestation scenarios, including infestation on insecticide-treated plants. Due to the fast speed of effect of the insecticide mixture product used (Verdavis), we expected a quick decrease in plant ES.

This study concludes with an experiment to compare ES recorded at different infestation durations and therewith resulting in a different plant damage severity. It furthermore compared EPE recordings from stem- and pod-inserted electrodes during the infestation and after manual stink bug removal. We hypothesized that stink bug infestation triggers long-distance plant ES that could be recorded from both plant stem and soybean pod. Finally, we hypothesized that the perpetuating plant damage due to the stink bug’s saliva does not result in the same plant ES as active stink bug feeding.

## Results

### EPE model development for stink bug infestation

**Fig. 1:**
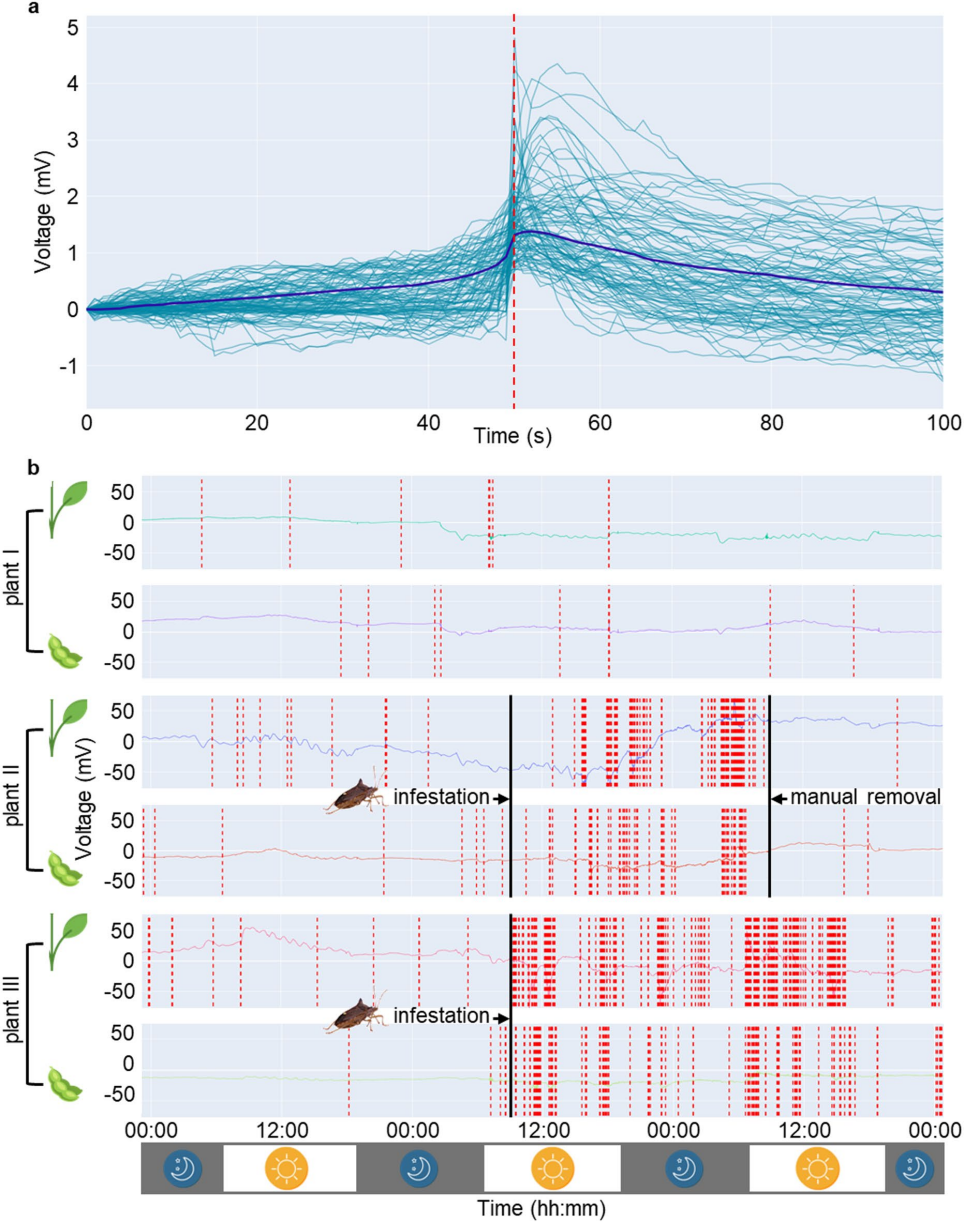
EPE recordings with spike detection model. **a** Synchronization of 100 close-up spikes in raw recordings (sky blue lines), average spike pattern (dark blue line) and EPE model spike detection (red dashed line). **b** 72-h EPE recordings (voltage in mV) show infestation and removal times from three distinct plants (recordings from ‘EPE model benchmarking’ experiment). Red dashed lines denote detected spikes; plant illustrations indicate electrode insertion in stem or pod.

An EPE model to detect plant ES was constructed based on the quantification of spike occurrences within the recorded signal (Fig. 1a). Signals were thereby first preprocessed, setting up a common baseline and a bandpass filtering (bandpass 0.010 to 0.0025 Hz). Spike identification of the preprocessed signal was then done with a 1 mV threshold. The model was iteratively optimized through expert supervision. Low amplitude spikes were most abundant but were affected by a decreased signal-to-noise ratio. The minimum threshold of 1 mV was chosen to maximize the differential spike count between infested and non-infested plants while maintaining a sufficient total spike count to preserve output resolution. Spikes outside the bandpass with higher frequencies (> 0.010 Hz) lowered quantitative output and showed less difference between treatments. Whereas lower frequencies (< 0.0025 Hz) resulted in an increased spike count but lowered treatment differences. Figure 1b illustrates the process of identifying spikes in raw electrical signals, using EPE recordings from electrodes placed in the stems and pods of three distinct plants. To improve homogeneity of variance and normality of error distribution, logarithmic transformation (log_10_ [x + 1]) was applied for all quantitative analyses of the model spike count.

### Stink bug infestation scenarios

**Fig. 2:**
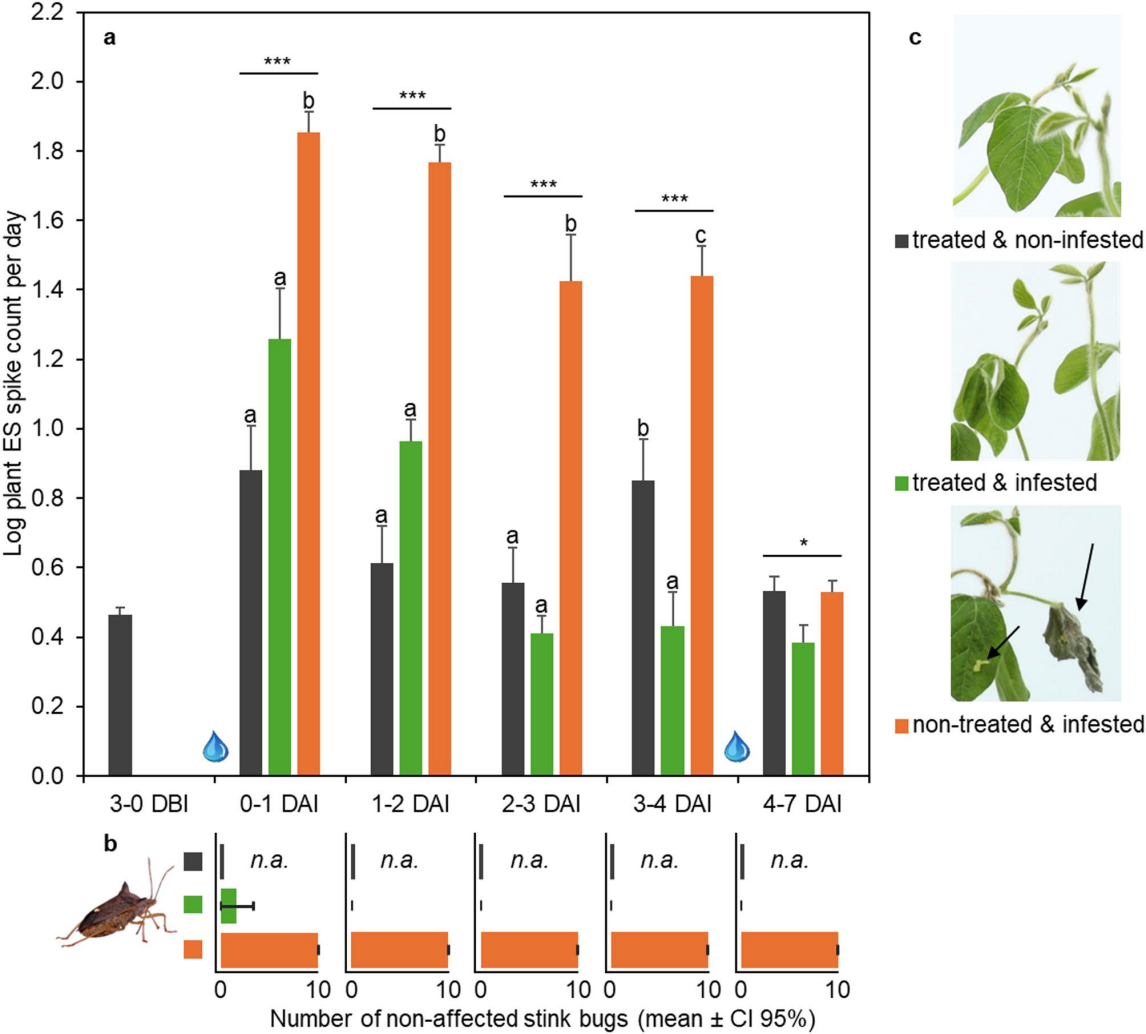
Early-season infestation scenario. VC-stage soybean plants (n = 6 pots) were sprayed with the insecticide and three days later infested with 10 *E. heros* adults per pot. **a** average number of spikes in plant electrical signals after raw data transformation; *, **, *** denote *p* < 0.05, 0.01, 0.001, error bars represent SE. DBI/DAI = days before/after infestation; water drops denote irrigation events. **b** count of non-affected stink bugs at the end of each time window; error bars represent CI 95%, n.a. = not applicable. **c** representative shoots of each treatment 7 DAI; arrows show stink bug damage and eggs.

In the early-season infestation scenario with VC-stage soybean plants, the EPE model output was significantly different between treatments (Fig. 2a). Infested plants that did not receive an insecticide treatment beforehand produced more spikes than non-infested plants or infested plants that were treated. This effect was already visible during the first day of infestation (F = 17.24, *p* < 0.001).

Interestingly, from the onset of infestation, insecticide-treated plants did not display statistically significant differences in their ES response compared to non-infested control plants. They appear to maintain this normalized state, even in the face of ongoing pest pressure. Furthermore, on the third day of infestation, the treated plants produced significantly fewer spikes than non-infested plants.

After the first irrigation, an increased fluctuation in spike detection was observed (black bars in Fig. 2a). This occurred despite precautions taken to minimize mechanical disturbance to both the plant and the quarantine system. Furthermore, an overall decline in spike count was observed in continuously infested plants, resulting in non-significant differences in the post-hoc test between treatments at the end of the experiment.

The insecticide mixture product provided rapid efficacy against stink bug adults on VC-stage soybeans. By one day after infestation (DAI), an average of 83% of stink bugs exposed to treated VC-stage soybeans were dead or affected (Fig. 2b). At 2 DAI, 100% of the stink bugs were dead or affected. In contrast, on non-treated control plants, all stink bugs survived and exhibited normal behavioral patterns throughout the experiment. Confidence intervals for the number of non-affected (visual assessment) stink bugs on treated VC-stage soybeans did not overlap with those on non-treated soybeans.

In line with the rapid efficacy on treated plants, no plant damage was visible to the naked eye (Fig. 2c). This implies that the stink bugs categorized as non-affected at 1 DAI on treated soybeans did not cause visible plant damage. In contrast, stink bug infestation on non-treated VC-stage plants resulted in moderate feeding damage. Especially shoot tips were affected on these plants, hampering normal plant growth. Furthermore, adult females deposited eggs on young leaves, potentially leading to subsequent feeding damage through second-generation nymphs.

**Fig. 3:**
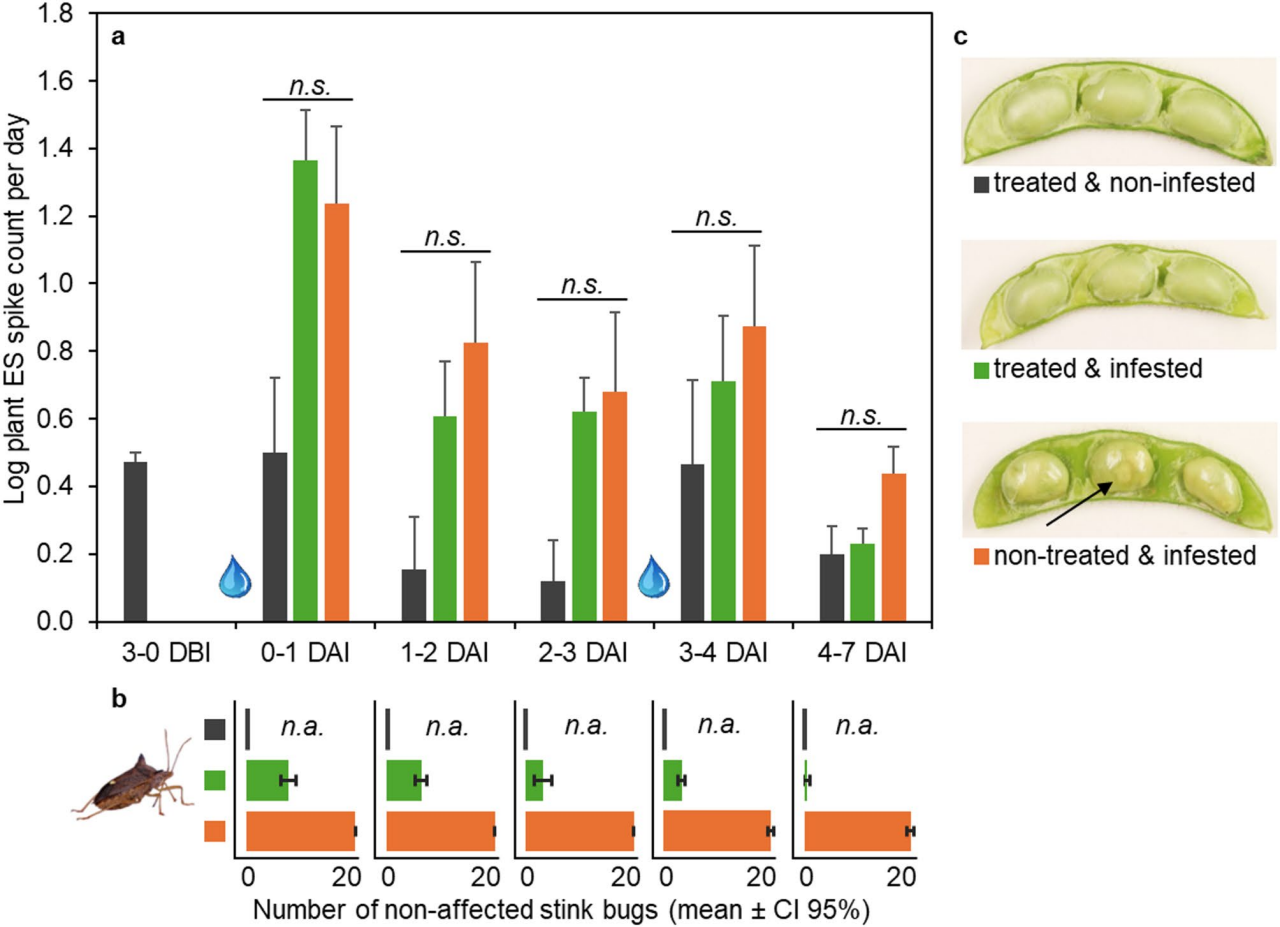
Late-season infestation scenario. R5-stage soybean plants (n = 5 pots) were sprayed with the insecticide and three days later infested with 20 *E. heros* adults per pot. **a** average number of spikes in plant electrical signals after raw data transformation; n.s. denotes non-significant for *p* < 0.05, error bars represent SE. DBI/DAI = days before/after infestation; water drops denote irrigation events. **b** count of non-affected stink bugs at the end of each time window; error bars represent CI 95%, n.a. = not applicable. **c** representative soybean pods of each treatment 7 DAI; arrow shows stink bug damage.

The late-season infestation scenario with R5-stage soybean plants was characterized by a reduced spike count compared with the other experiments (Fig. 3a). This low spike count resulted in no statistically significant differences between treatments at all time intervals (e.g., during the first day of infestation F = 0.66, *p* = 0.058). The observed electrophysiological patterns were consistent with the minimal visible plant damage documented at the end of the experiment (Fig. 3c). In agreement with the preceding experiment, a temporal variability increase after irrigation, but overall general decline in spike count was observed across all treatment groups (Fig. 3a).

The insecticide demonstrated rapid efficacy against adult stink bugs on R5-stage soybeans, similar to its effect on VC-stage soybeans. By 1 DAI, 62% of stink bugs subjected to treated plants were dead or affected (Fig. 3b). The apparent insecticidal effects increased gradually over time, with the proportion of visually affected and dead stink bugs reaching 98% by 7 DAI. In contrast, on non-treated control plants, the majority of stink bugs survived until the end of the experiment (i.e., categorized as non-affected). Confidence intervals for the number of non-affected stink bugs on treated plants did not overlap with those on non-treated soybeans at any evaluation timepoint.

None of the R5-stage plants showed clear visual signs of feeding damage. Upon opening of soybean pods, minor damage was visible on non-treated infested beans (Fig. 3c). No damage was visible in pods of treated plants aligning with rapid efficacy. These results imply that stink bugs categorized as non-affected between 1 and 7 DAI after exposure to treated plants did not engage in substantial feeding activity.

### EPE model benchmarking

**Fig. 4:**
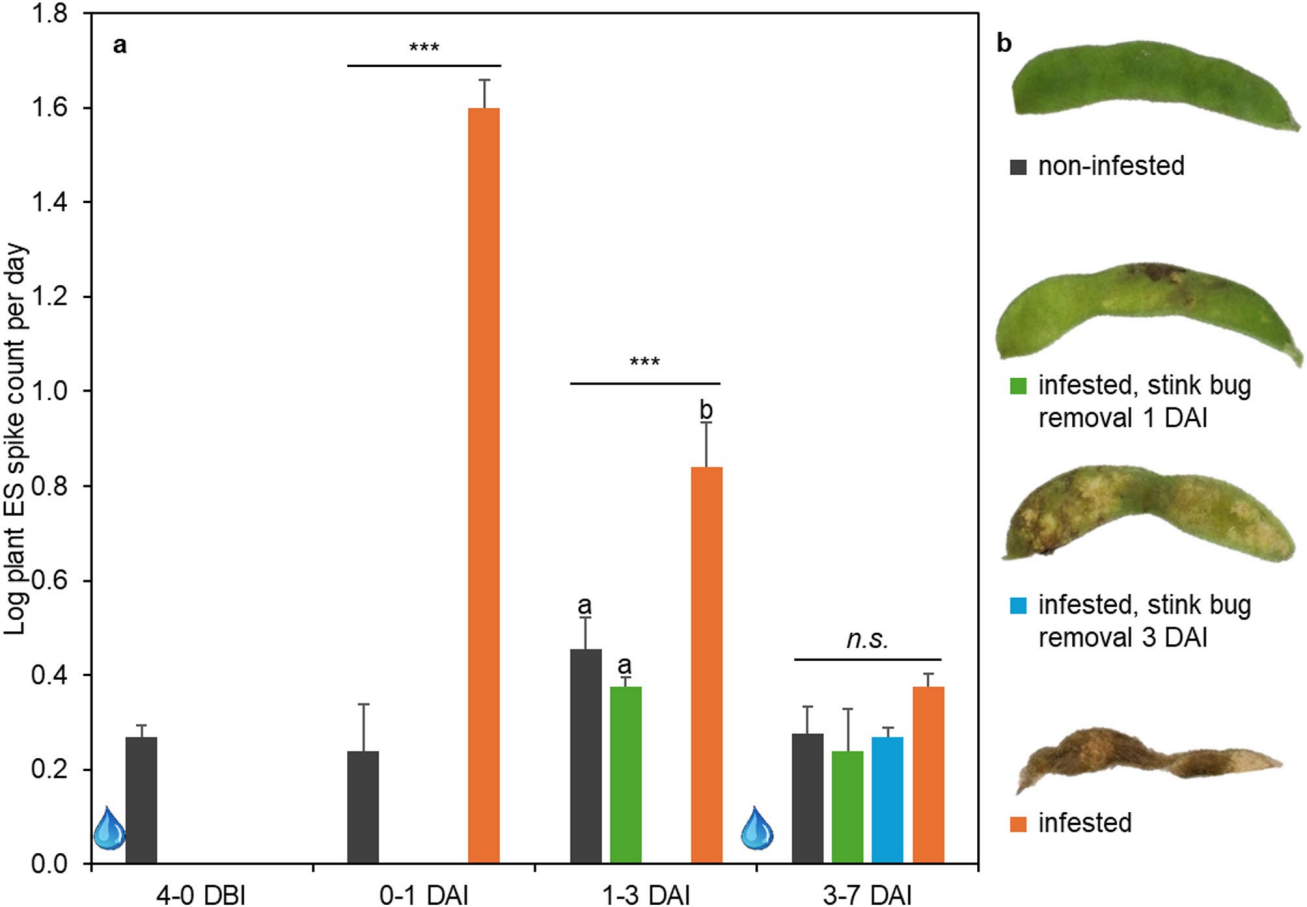
EPE model benchmarking. R5-stage soybean plants (n = 4 pots) were infested with 50 *E. heros* adults per pot. **a** average number of spikes in plant electrical signals after raw data transformation; n.s. denotes non-significant and *, **, *** denote *p* < 0.05, 0.01, 0.001, error bars represent SE. DBI/DAI = days before/after infestation; water drops denote irrigation events. **b** representative soybean pods of each treatment 7 DAI.

In the EPE spike model benchmarking experiment, ES were recorded from pod- and stem-inserted electrodes. Figure 4a shows EPE spike counts that are averaged over both electrode insertion types from the same plants. Plants subjected to stink bug infestation exhibited a statistically significant increase in spike generation frequency compared to non-infested controls until 3 DAI. Upon manual removal of the stink bugs at 1 or 3 DAI, the spike generation rates of previously infested plants reverted to levels comparable to those observed prior to infestation. Furthermore, like in the other two experiments, plants subjected to continuous infestation demonstrated a decline in spike count over time.

Throughout this experiment, all stink bug adults survived and exhibited normal behavioral patterns on the R5-stage soybeans. Plant damage of soybeans infested with stink bugs gradually increased with the duration of infestation (Fig. 4b). Soybeans from which stink bugs were removed 1 DAI exhibited moderate feeding damage to pods (seed damage < 30%). Damage increased to severe pod damage (seed damage 51–70%) when stink bugs were removed 3 DAI. After seven days of continuous stink bug infestation, soybean pods were dead (seed damage > 71%), showing complete destruction and discoloration.

Making use of the high number of infested plants during this time, a more detailed analysis was conducted on the recordings of the first day of infestation. The EPE spike model did not reveal a statistically significant difference in spike count between pod- and stem-inserted electrodes during daytime (i.e., light) (t = 0.21, *p* = 0.837) or during nighttime (i.e., darkness) (t = 1.02, *p* = 0.328). However, weighed per hour, more spikes were obtained during the day than during the night (t = 5.11, *p* < 0.001). Using raw (non-transformed) hourly data, the median spike count was 3.7 times higher during the day compared to night. Spike amplitudes followed a normal distribution and did not require data transformation. The overall spike amplitude average and standard deviation of infested plants during this period were 1.9 and 0.8 mV. Spike amplitudes were similar between stem- and pod-inserted electrodes (t = 0.99, *p* = 0.345), and between day and night recordings (t = 0.01, *p* = 0.989).

## Discussion

Adult *E. heros* successfully infested soybean plants during early growth (VC-stage) and pod filling (R5-stage) phenology, causing serious damage to plant shoots and pods. The last two experiments with R5 plants showcased stink bugs’ complex and discontinuous feeding behavior, necessitating an analysis of both pest insect infestation and related host plant stress.

In the early-season infestation scenario (Fig. 2), the EPE model output for plants treated with the insecticide mixture product of isocycloseram and lambda-cyhalothrin showed no statistically significant difference from non-infested plants shortly after infestation started. Frequent assessments of stink bug mortality revealed that the majority of the pest population was effectively controlled within 24 h of infestation. Over the subsequent days, the infested non-treated plants continued to produce significantly more spikes. The EPE output statistically aligned well with crop damage symptoms visible at the end of the experiment, whereas EPE captured this already during the first four days of infestation.

The late-season infestation scenario using R5-stage soybean plants (Fig. 3) resulted in unexpectedly low visual feeding damage. The reason for this remains unclear, particularly as the EPE-benchmarking experiment documented predominant pod feeding damage. Conversely, stink bugs were allowed more freedom of movement off the treated plant in the R5-stage cages than in the VC-stage plant containment. The quarantine system used for R5-plants was 40 times larger compared with containers of the VC-stage experiment. This potentially reduced their chance for exposure to the treated R5-stage plant. Stink bugs’ variability in general and feeding behavior is well studied in literature^13^. In other studies, such variable behavior was attributed to the relatively short feeding events needed on the highly nutritious pods, from which *E. heros* prefers to feed^29^. The authors found a comparably long feeding time for *Edessa meditabunda* (Hemiptera: Pentatomidae), demonstrating long feeding bouts of low nutritious stem feeding. As seen in Fig. 3, a conventional interval-based visual assessment of live stink bugs cannot capture the temporal and spatial distribution of stink bugs on plants. On the other hand, EPE results identified minimal presence on plants from the first day of infestation, which was later confirmed by the absence of notable visual plant damage seven days after infestation.

In the EPE-benchmarking experiment (Fig. 4), the ES spike count decreased to levels comparable to those of non-infested plants, one day after manual removal of insects. Notably, infestation duration, and consequently plant damage, did not influence the frequency of spikes generated after infestation stop. The spike count dropped over time, reaching non-significantly different levels between three and seven days after infestation. Additionally, this experiment highlighted the severe impact that *E. heros* infestation can have on soybean pod quality. Under this high infestation pressure, significant pod deterioration was evident already after a one-day infestation period. Complete yield loss occurred after seven days of continuous infestation. Therefore, crop protection measures need to provide successful stink bug feeding control within the first day after application. Lastly, EPE output again aligned well with visual plant damage.

EPE responses to insect infestation offer promising opportunities for the earliest detection of crop damage. However, insect-induced responses still need to be distinguished from other types of similar plant responses within agronomically relevant conditions. The results of our experiments indicate that the EPE model output was influenced by factors beyond stink bug infestation.

A general decrease in spike detection was observed over time, partially offset by temporary increases following irrigation events. This pattern may firstly be attributed to the plant’s inherent capacity to generate spikes or its stress adaptation mechanisms, lowering spike generation. Secondly, changes in the sensitivity of inserted electrodes to perceive plant ES in their vicinity may decrease due to walling off of electrodes^30^. Watering expands stem tissue and increases electrical conductivity. Thirdly, other types of plant ES that are picked up by the EPE model, for example related to watering, may temporarily increase spike detection. However, the detected spikes differed greatly from water status-related EPE patterns found in other studies using this technology^25^. The experiments demonstrating the most severely damaged tissue, i.e., the soybean pods in the EPE-benchmarking experiment, also exhibited the most significant decrease in spike count over time (visible for stem- and pod-inserted electrodes). This correlation suggests that the extensive tissue damage may have compromised the local plant tissue’s capacity to generate and convey ES.

Research comparing plant responses to different pest species over time found that the intensity, start time, and stop time of plant ES generation are pest-specific^31^. Even within the same plant–insect setup, the duration of plant cells’ excitability for ES is highly variable and situational^12^. Moreover, our results show for plants with visible damage, the frequency of electrical spikes was higher during daylight hours compared to nighttime. These results suggest a light-darkness-dependent or circadian regulation of plant reaction to biotic stress^32^. Additionally, water availability influences this interplay by affecting the jasmonic acid pathway, which is modulated by ES^33,34^. Besides having a direct effect, it is therefore possible that irrigation events indirectly impact infestation-related plant ES. Experimental scenarios whereby light conditions are altered independently from daily rhythms might help disentangle insect, plant and environment interactions.

In the EPE benchmarking experiment with R5-stage plants, once stink bugs were removed, the EPE model did not show an elevated number of plant ES, compared to non-infested plants. Conversely, in the early-season scenario with VC-stage plants, an elevated spike count was seen for three days after feeding stop. In the absence of soybean pods, stink bug feeding on the VC-stage plants concentrated on the shoot apex. The disruption of primary stem growth might create a plant ES response that perpetuates after infestation stopped. Stem feeding and its effect on plant homeostasis has a major influence on plant survival, especially for young plants^35^. Indeed, the infestation of non-treated plants resulted in dead shoot tips and young leaves, completely halting plant growth.

The EPE spike model may detect ES specifically associated with the lingering activity of digestive enzymes in the stink bug’s saliva. Furthermore, plant damage established itself over several days after infestation stopped, acting as a potential source for continued ES generation. Research on *Nezara viridula* (Hemiptera: Pentatomidae) soybean seed feeding demonstrated that salivary enzymes (including hydrolases and oxidoreductases) were more important than mechanical damage, to induce an infestation-specific plant response^36^. Comparative studies involving *Spodoptera littoralis* (Lepidoptera: Noctuidae) feeding and mechanical damage have clearly demonstrated the importance of insect oral secretion elicitors in generating plant ES^23,37^. These studies highlight that plant ES is among the initial responses to herbivory, subsequently triggering downstream reactions such as phytohormone production and metabolic changes.

Soybean plants respond to stink bug infestation by altering MAP-kinase expression within 10 to 20 min, while jasmonic acid levels peak after three hours^34,38^. Research on the protein profile of the saliva of several stink bug species revealed that salicylic and jasmonic acid-dependent plant defense may possibly be suppressed^39^. A suppressed plant reaction may be one of the reasons why a decreased spike count was visible during the latter part of the experiments. Conversely, as shown in Fig. 1b, immediate ES spike generation is visible upon infestation, with no apparent decline over time during the first 24 h.

Plant genomes contain a multitude of genes encoding putative transmembrane proteins with potential importance in ion transport. The established classification of long-distance plant electrical signals includes primarily action and variation potentials (i.e., slow wave potentials), which are induced by non-damaging and damaging stimuli respectively^15,40^. Action and variation potentials are typically characterized by amplitudes ranging from 20 to 100 mV and transmission rates of several cm per minute^21^.

In our results, insect-related electrical potential changes were typically lower than 5 mV and were detected 20 to 80 times per plant during the first day of infestation. This high occurrence rate is probably the result of the multitude of sources, each inflicting damage at different tissues and time. Conversely, the lowered signal amplitudes may be the result of too numerous excitations propagating across long distances. This may finally lead to subthreshold responses (in case of action potentials). In case of metabolites that function as propagation mechanism for variation potentials, this may lead to distinct transmission patterns^21^.

Low-amplitude electric potential differences may be attributed to several factors. Firstly, these may be considered as sub-threshold electric potential variations. However, it raises the question of how these signals propagate throughout the plant without the aid of a cellular depolarization wave. Secondly, spikes may be related to tissue-specific depolarizations^41^. When such action potentials are limited to specific plant tissues, they merely constitute a small portion of the total electrochemical field surrounding the electrode. Consequently, the overall electrode recording output would result in only minor amplitude changes. A comparison of ES recordings from various tomato (*Solanum lycopersicum*) tissue types after wounding clearly demonstrated this. ES recordings in sieve-tube elements and companion cells exhibited a four-fold higher amplitude compared to other cell types^42^.

Alternatively, the ES measured with EPE may originate from hydraulic pressure waves, as described for variation potentials. The variable amplitude of the recorded ES, although potentially influenced by signal preprocessing, is characteristic of pressure-based ES^15^. However, the short spike duration (i.e. repolarization phase) and low amplitude observed in our study are atypical for variation potentials^24^. Additionally, the overall consistent count and amplitude of spikes measured by both pod- and stem-inserted electrodes from the same plant are unusual for this type of ES. Spike counts were equally high at both electrode locations (EPE-benchmarking experiment) and when electrode position was distant from visual plant damage on the shoot tip (early-season infestation scenario). However, to elucidate the exact nature of these plant ES, dedicated experiments with distinguishable single stress events at different plant organs, and knockout plant mutants that prevent specific plant ES pathways^37,43^ would be beneficial.

Previous research using extracellular measurements of ES outside a Faraday cage has demonstrated the ability to distinguish plants under both abiotic^25^ and biotic^28^ stress in real-world settings. While these studies are of high agronomic importance, they relied on black box machine learning models to differentiate between stressed and non-stressed plants. In our study, we successfully employed the quantification of filtered ES to identify plant signals related to stink bug infestation. These plant ES differed greatly from drought stress^25^ and nematode (*Meloidogyne incognita*) infection^28^ experiments conducted with the same type of recording devices. Mathematical models provide clear quantifiable output. This helps to compare signal output between experiments with different designs and enables scaling based on infestation level and plant damage thresholds. Although we tested our model with a single stink bug species, the model could likely be implemented for other pests that provoke similar plant response like plant and leaf hoppers.

In summary, these experiments show that the developed EPE model is applicable across different plant phenological stages and plant tissues for electrode insertion. The model’s sensitivity range incorporated spike detection from beginning plant damage up to the point of total loss of plant organs. This study showcases another example that EPE is a promising non-invasive, real-time method for monitoring plant stress responses to herbivory^28^. The technology enables remote monitoring and infestation detection before visible damage occurs. Further research will be directed to field recordings throughout a growing season, and the model’s response to other biotic and abiotic stresses.

## Materials and methods

Soybeans (*G. max*) cv. Toliman were grown in a mixed soil with fertilizer under the same greenhouse conditions as during the experiment. Depending on the experiment, soybeans were grown until VC-stage (vegetative cotyledon, expanding of first unifoliate leaf) or R5-stage (start of seed development in soybean pods)^44^. R5-stage plants were grown individually in plant pots, whereas VC-stage plants grew in sets of four plants per pot to ensure sufficient food substrate for stink bugs.

Treated soybean plants received a preventative foliar application of 62.5 g active ingredient ha^-1^ Verdavis ZC250 (Syngenta Crop Protection, Basel, Switzerland). The application was conducted with 500 l ha^-1^ spray volume, using a turntable device for 360-degree spray coverage. Verdavis ZC250 consists of 150 g l^-1^ lambda-cyhalothrin (IRAC group 3: pyrethroids) and 100 g l^-1^ isocycloseram (Plinazolin technology) (IRAC group 30: isoxazolines)^45–48^. All other treatments received a water application of equal volume. The application was carried out three days before infestation.

Neotropical brown stink bugs (*E. heros*) were reared until adulthood on soybean plants and were offered fresh pole beans (*Phaseolus vulgaris*) and crushed peanuts (*Arachis hypogaea*) as additional food. Rearing was conducted at 25 °C, ca. 60% relative humidity and a 16-h light, 8-h dark photoperiod. A cohort of newly emerged adults from a synchronized developmental batch of stink bugs (39 days after oviposition) were used in experiments with stink bug infestation. For the infestation process, insects were positioned adjacent to individual soybean plant pots. Each pot was contained within its own quarantine system to prevent cross-contamination. For VC-stage plants, 5-L plastic cylinders served as containment units, restricting stink bug mobility to the plant and soil environment. In contrast, R5-stage plants were housed in 216-L enclosures (60 × 60 × 60 cm cages), permitting insect movement both on the plant and around the surrounding pot area. The infestation intensity differed per experiment (Table 1). Manual removal of stink bugs during the final experiment was done by hand.

**Table 1:** Comparative overview of the stink bug infestation experiments.

	Early season infestation	Late season infestation	EPE model benchmarking
Plant growth stage	Vegetative (VC)	Reproductive (R5)	Reproductive (R5)
Number of pots per treatment (n)	6	5	4
Number of stink bugs per plant pot	10 adults	20 adults	50 adults
Position of active electrode	Stem	Pod	Stem/pod
Position of ground electrode	Stem	Stem	Stem/stem
Time of electrode insertion	3 DBI	3 DBI	4 DBI
Stink bug infestation stop due to	Insecticide	Insecticide	Manual removal

DBI = days before infestation.

Experiments were conducted under controlled greenhouse conditions. The environment was maintained with a 14-h day and 10-h night photoperiod. Temperatures were regulated at 24 °C during the day and 20 °C at night, with relative humidity held constant at ca. 60%. Plant irrigation was applied uniformly and performed on demand to prevent drought stress. Specific watering events are provided in the corresponding results figures. Plants were watered until field capacity.

Plant ES were measured extracellularly and outside a Farraday cage, using Biosignals recording devices (Vivent SA, Gland, Switzerland)^28^. Briefly, two electrodes that were made of 5 mm long silverplated stainless steel needles (0.25 mm in diameter) were inserted into the plants. Using coax cables (about 1 mm in diameter, 50 Ω) with a silver coated copper wire as inner conductor and a waterproof copper outer conductor, the electrodes were connected to a recording device that measured electrical potential difference.

**Fig. 5:**
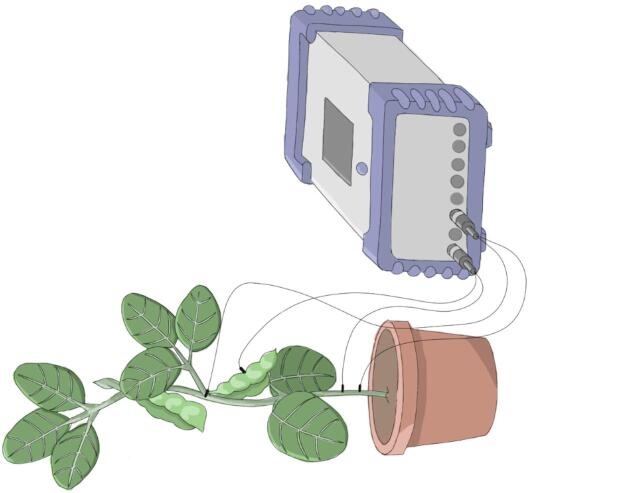
Plant ES recording with EPE. Illustration of the EPE setup with Vivent Biosignals recording device and electrode insertions into soybean pod and stem (drawing provided by Nicolas Marguier).

Each recording circuit consisted of an active and a ground electrode, inserted into the shoot top and base, respectively, for stem-inserted electrodes. Stem-inserted electrodes were pierced through the center of the stem, i.e., penetrating vascular bundles. Tips were able to completely penetrate the stem, and active and ground electrodes were inserted from opposite sides into the plant. For pod-inserted electrodes, the active electrode was completely (i.e., 5 mm) pierced ventrally into the pod locule (Fig. 5). Electrodes were inserted three to four days before infestation to minimize wound damage response in ES recordings and to allow plant recovery. The lightweight electrodes and cables did not cause detectable mechanical wound damage during infestation.

In total, three greenhouse experiments were conducted. The first two experiments simulated infestation scenarios on insecticide-treated and non-treated plants, whereas the final experiment was based on manual removal of stink bugs after specific time intervals (Table 1).

The electrodes were inserted after the preventative application, i.e. three days before infestation, in the early and late season infestation scenarios. Subsequently, the number of non-affected, as well as dead and affected insects were counted one, two, three, four and seven days after infestation. Stink bugs were classified as ‘affected’ when they exhibited obvious signs of impairment, including leg paralysis, ataxia, and an inability to maintain normal posture. These symptoms result in stink bugs being in supine or lateral positions, rendering them incapable of feeding on and thus causing damage to the host plant.

In the EPE model benchmarking experiment, ES from the same soybean plant were recorded from a stem- and a pod-inserted electrode in parallel. EPE recordings started four days before stink bug infestation, and the infestation level was increased to 50 adults per plant to ensure severe crop damage. In two treatments stink bugs were manually removed after one or three days of infestation. The final treatment was kept under constant infestation for seven days.

**Fig. 6:**
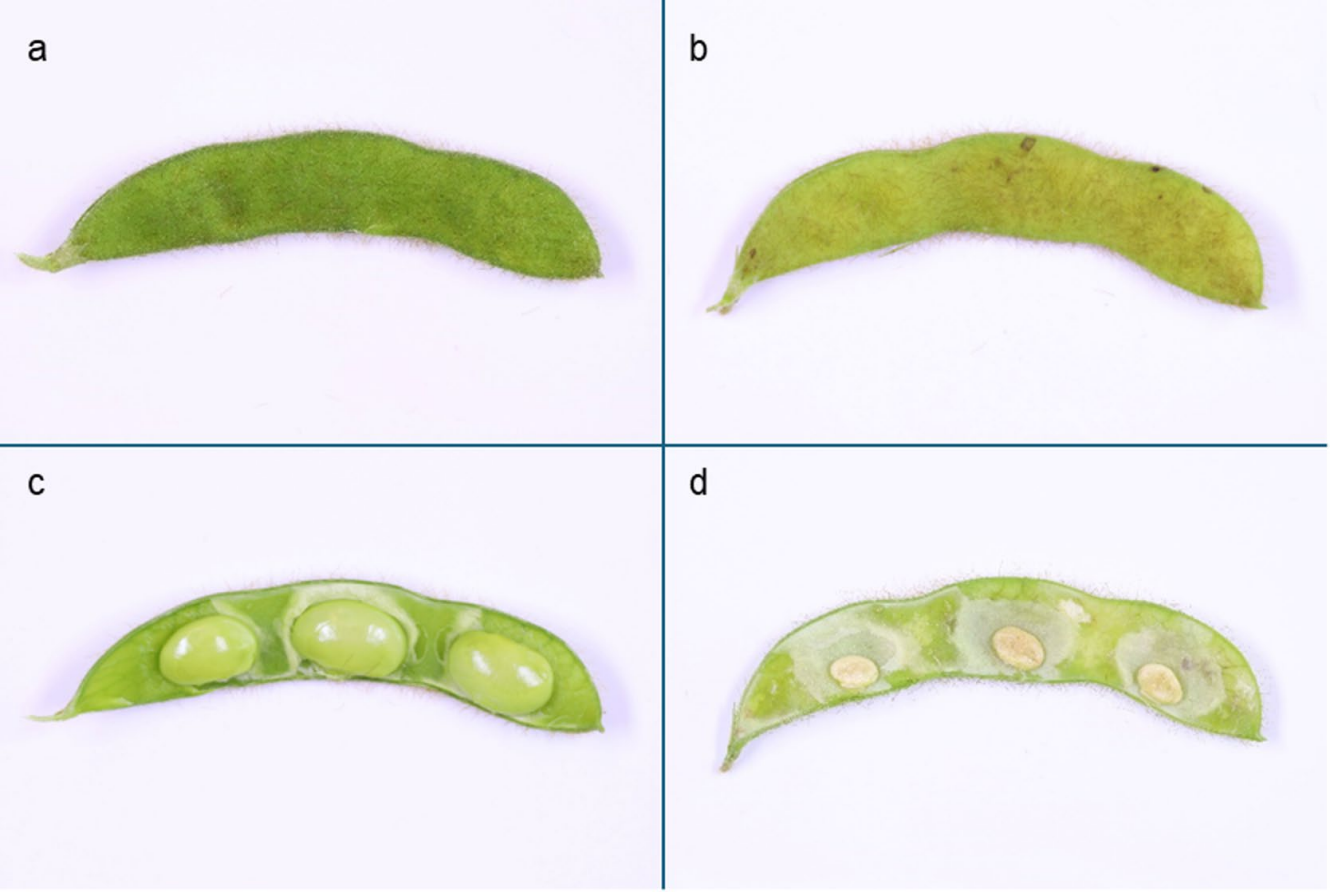
Illustration of stink bug feeding damage on soybean pods. Closed (**a**, **b**) and opened (**c**, **d**) soybean pods. **a**,** c** demonstrate a healthy pod and **b**, **d** a stink bug-damaged pod. Pods from non-infested plants reached the developmental stage R6 (full seed development) at the end of the experiment whereas pod development on infested plants was halted at R5 (beginning seed filling) and their pods showed tissue discoloration due to stink bug feeding activity.

At the end of each experiment, i.e. after up to seven days of infestation, the plant damage was visually assessed. Soybean pod damage was quantified by percentage of seeds damaged and/or aborted. Pods with an average of 1–30%, 31–50%, 51–70% and 71–100% damaged seeds were classified as moderate, critical, severe, and complete (i.e., dead pods) respectively. Depending on the damage level, soybean pods were opened ventrally for a more differentiated assessment (exemplified in Fig. 6). Seed damage can lead to seed abortion, delaying visual damage symptoms.

Statistical analyses were performed with SAS V9.4 (SAS Institute, Cary, North Carolina). Efficacy data after insecticide application were descriptively analyzed by plotting means with 95% confidence intervals. This approach was chosen since there was hardly any stink bug mortality without insecticide application. EPE data were analyzed with an analysis of variance, followed by Tukey’s post-hoc test, or using paired t-tests across the same electrode or plant.

## Data Availability

The datasets generated during and/or analyzed during the current study are available from the corresponding author on reasonable request.
